# Supplementary material for: Identification of Critical Genes and Pathways for Influenza A Virus Infections via Bioinformatics Analysis
Source: Viruses. 2022 Jul 26;14(8):1625. doi: 10.3390/v14081625 (PMC9332270; doi:10.3390/v14081625)
Supplement: Supplementary file 1 [file viruses-14-01625-s001.zip › Table S1. The selected samples from 8 GEO datasets in this analysis.pdf]

Table S1. The selected samples from 8 GEO datasets in this analysis

Datasets	Size	The selected samples
GSE165340	6	GSM5031744- GSM5031749
GSE97672	16	GSM2575117- GSM2575132
GSE104168	18	GSM2791029- GSM2791037; GSM2791044- GSM2791052
GSE156152	6	GSM4725672- GSM4725677
GSE163959	64	GSM4991245- GSM4991108
GSE193164	6	GSM5775734; GSM5775735; GSM5775738; GSM5775739; GSM5775742; GSM5775743
GSE186908	162	GSM5663456- GSM5663617
GSE89008	20	GSM2357098- GSM2357117
