# Supplementary figures and images for: Identification of Critical Genes and Pathways for Influenza A Virus Infections via Bioinformatics Analysis
Source: Viruses. 2022 Jul 26;14(8):1625. doi: 10.3390/v14081625 (PMC9332270; doi:10.3390/v14081625)

# Batch corrected

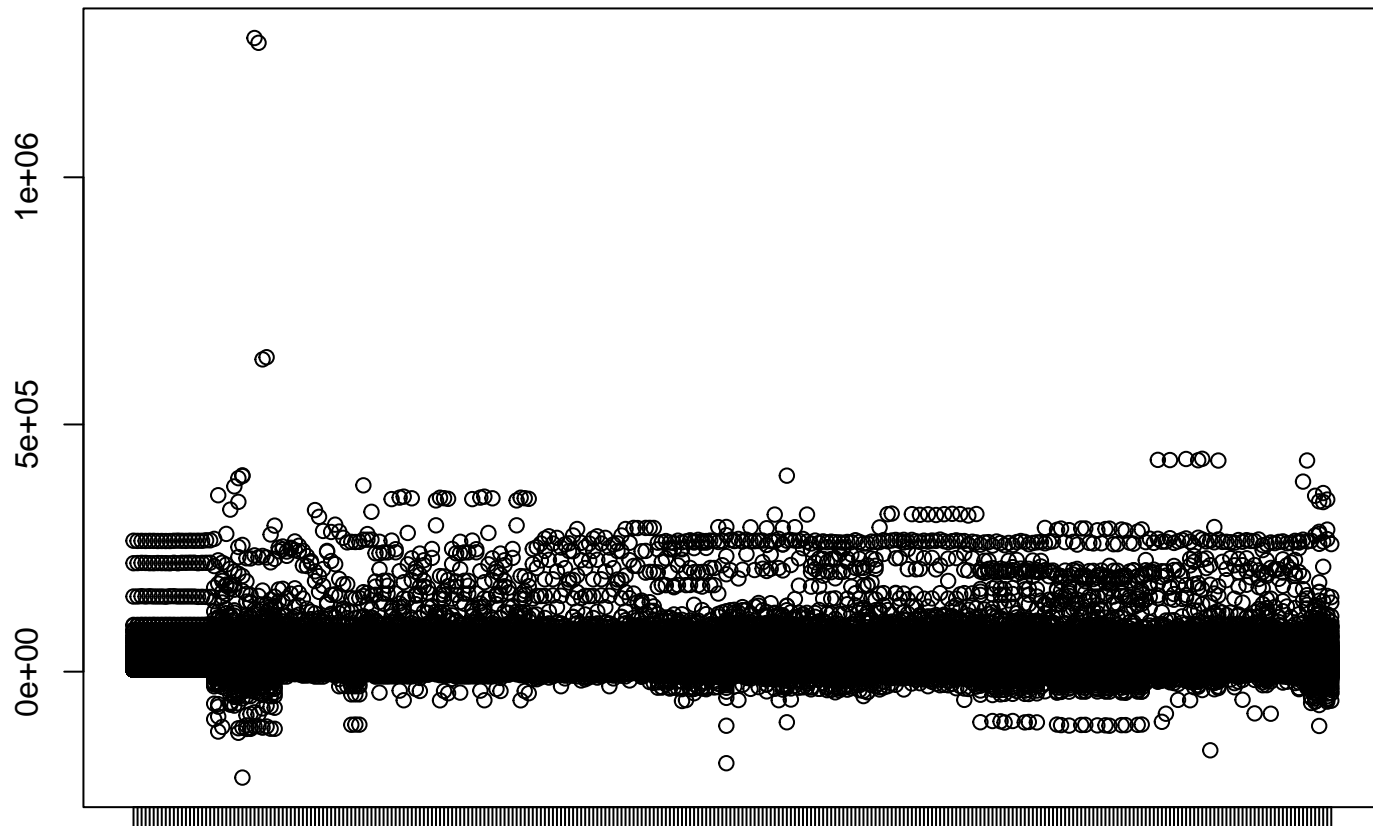

HTBE\_H1N1\_MOI5\_03h\_1 norm.L18 MOCK.2 s2\_A8 s2\_C3 s1\_D9 s2\_F3 s1\_G7

Supplement: Supplementary file 1 [file viruses-14-01625-s001.zip › Figure S1. The boxplot of batch-corrected data.pdf]

### Scale independence

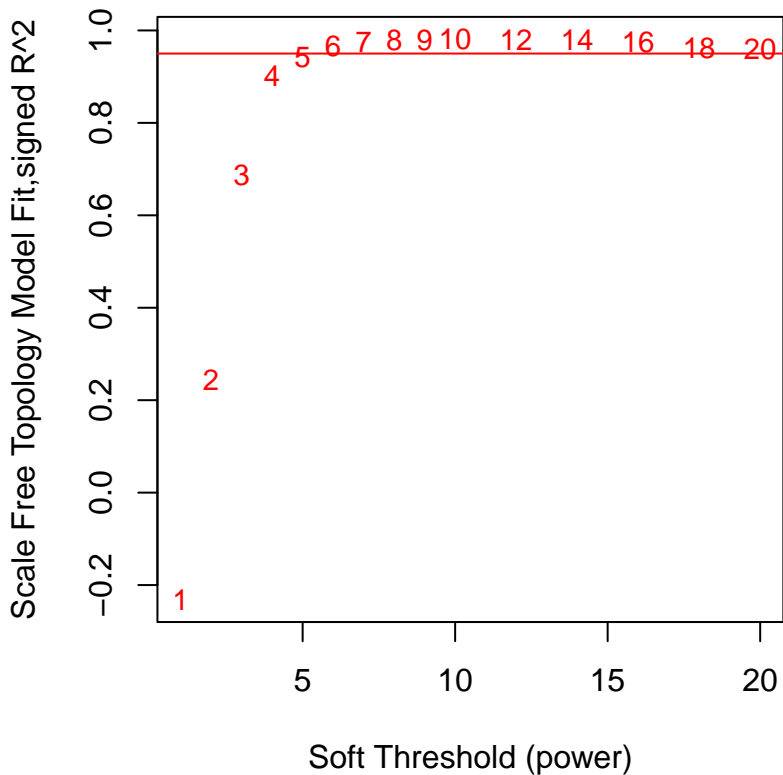

### Mean connectivity

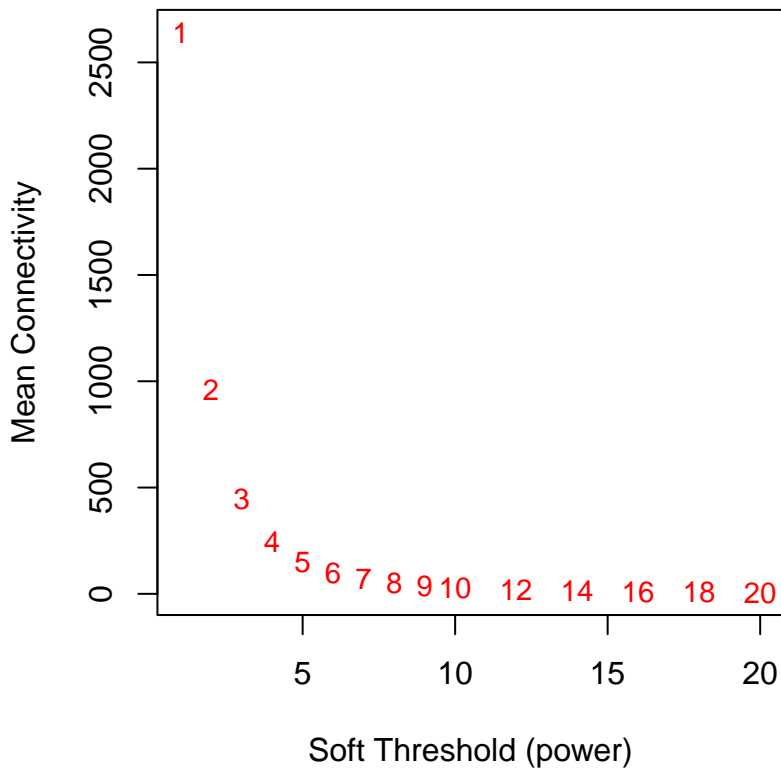

Supplement: Supplementary file 1 [file viruses-14-01625-s001.zip › Figure S2. Determination of soft-thresholding power..pdf]

# Module-trait

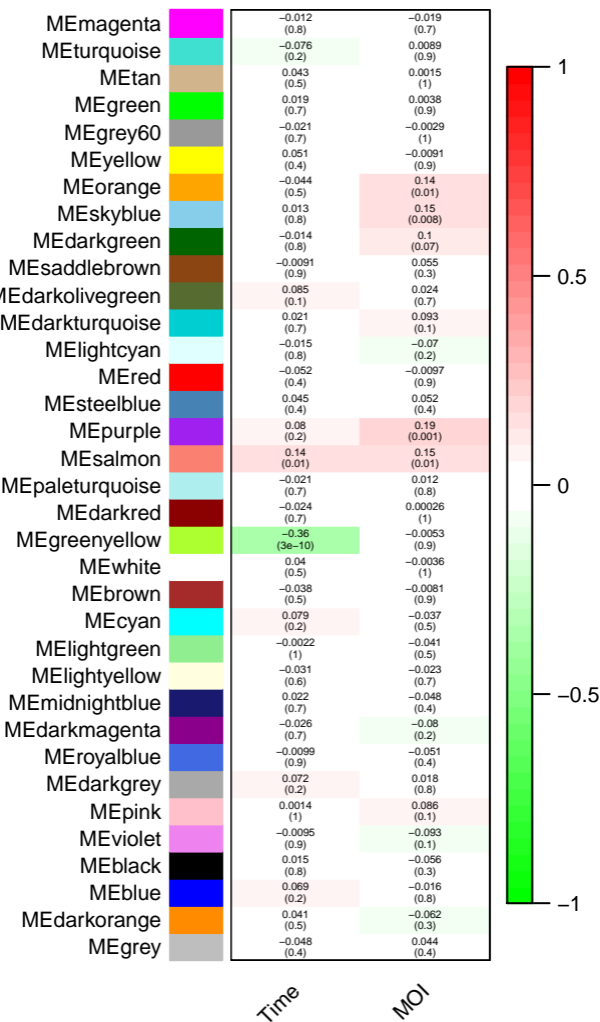

Supplement: Supplementary file 1 [file viruses-14-01625-s001.zip › Figure S3. The heatmap of relationships between module eigengenes and traits.pdf]
